# Climate Change, Health, and Vulnerability in Canadian Northern Aboriginal Communities

**DOI:** 10.1289/ehp.8433

**Published:** 2006-07-11

**Authors:** Christopher Furgal, Jacinthe Seguin

**Affiliations:** 1 Nasivvik Centre for Inuit Health and Changing Environments, Public Health Research Unit, Centre hospitalier Universitaire du Québec –Centre hospitalier Université Laval, Department of Political Science, Laval University, Québec City, Quebec, Canada; 2 Climate Change and Health Office, Health Canada, Ottawa, Ontario, Canada

**Keywords:** Aboriginal, adaptive capacity, Arctic, climate change, Inuit, vulnerability

## Abstract

**Background:**

Canada has recognized that Aboriginal and northern communities in the country face unique challenges and that there is a need to expand the assessment of vulnerabilities to climate change to include these communities. Evidence suggests that Canada’s North is already experiencing significant changes in its climate—changes that are having negative impacts on the lives of Aboriginal people living in these regions. Research on climate change and health impacts in northern Canada thus far has brought together Aboriginal community members, government representatives, and researchers and is charting new territory.

**Methods and Results:**

In this article we review experiences from two projects that have taken a community-based dialogue approach to identifying and assessing the effects of and vulnerability to climate change and the impact on the health in two Inuit regions of the Canadian Arctic.

**Conclusions:**

The results of the two case projects that we present argue for a multi-stakeholder, participatory framework for assessment that supports the necessary analysis, understanding, and enhancement of capabilities of local areas to respond and adapt to the health impacts at the local level.

There is strong evidence that Canada’s North is already experiencing significant changes in its climate (e.g., McBean et al. 2005). The climatic and environmental changes that have been observed during the last century require greater understanding and involvement by individuals and institutions to define effective adaptation strategies. Through signing the 1992 United Nations Framework Convention on Climate Change (2006) and ratifying the Kyoto Protocol (2006), Canada has shown its commitment to the global effort to slow the rate of warming, reduce emissions, conduct research, and initiate action at the national and regional levels to develop adaptation strategies to minimize the impact throughout the country (Government of Canada 2003). Canada has recognized that Aboriginal and northern communities face unique challenges and that it is necessary to expand the assessment of vulnerabilities to effects of climate change to all areas of Canada, including the North (Government of Canada 2003). This work is essential for the development of effective adaptive strategies to protect the health of Canadians in all regions of the country.

Assessing the impacts that these climate changes are having or may have on peoples’ lives requires a combination of disciplinary approaches and methods (Patz et al. 2000). Research on climate change and health impacts in northern Canada is in its infancy (Furgal et al. 2002). It uses and focuses particularly on indigenous knowledge and local observations of environmental change along with scientific assessments of the impacts associated with these and other forms of change. In this article we review experiences from projects that used a community-based dialogue-oriented approach to identifying and assessing potential health impacts and vulnerabilities to climate change in two Inuit regions of Canada’s North. These experiences build a strong case for a multi-stakeholder, qualitative, and participatory approach to identifying and assessing risks while enhancing the capacity of local areas to respond to the impacts of climate change.

## The Canadian North

**Figure 1:**
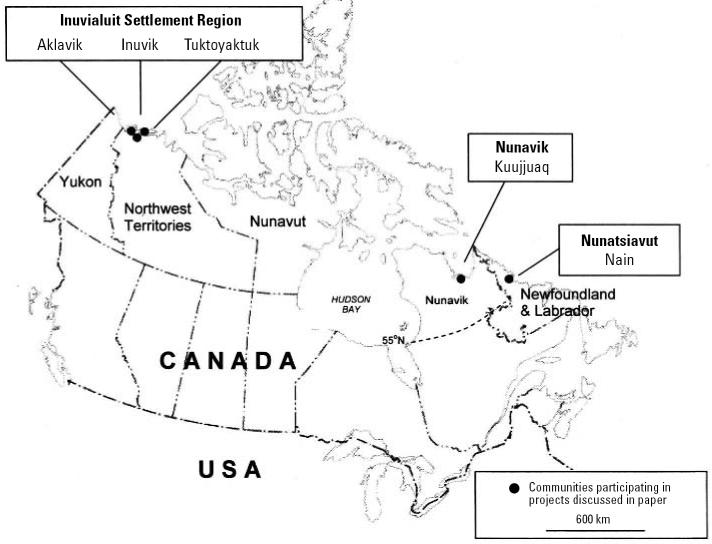
Map showing territories and regions of the Canadian North. Communities engaged in projects such as those discussed in Furgal et al. (2002), Nickels et al. (2002), and in this present article are identified.

A common definition of Canada’s North that we use here includes the three territorial administrative regions north of 60° latitude (Yukon, Northwest Territories, and Nunavut) as well as the region of Nunavik, north of 55° in the province of Québec and the Inuit settlement region of Nunatsiavut within Labrador. The latter two regions comprise communities with large Aboriginal populations and share many biogeographic characteristics with the territorial Arctic. Together, this region covers approximately 60% of Canada’s landmass (Figure 1).

The vast coastline, islands, and permanent multiyear ice found in Canada’s North are rich in geography and biodiversity. The diversity of the regions’ ecosystems, climate and cultures forms a socioecologic collage across the top of the country (Canadian Arctic Contaminants Assessment Report II 2003). Communities are spread along Canada’s northern coastline and interior, and the land and sea provide northern residents with a primary source of nutrition and form a central part of their livelihoods and cultures (Van Oostdam et al. 2005).

Northerners have witnessed profound environmental, social, political, and economic changes in recent decades (Damas 2002; Wonders 2003). Research on contaminants, and more recently on climate change, has uncovered what many northerners have known for some time: the Arctic environment is stressed and irreversible changes are occurring. At the same time, many communities are transitioning economically, having become more permanent than they were 40 years ago. Many communities now have a mixed economy of traditional or land-based activities and wage employment, with many of the wage employment opportunities now associated with large-scale development of nonrenewable natural resources (e.g., mining). These increases in development and cash income have resulted in changes in local economies and increased accessibility to many market items typically available in urban centers to the south. Further, dramatic political changes have resulted in Aboriginal groups in many regions now leading regionally based forms of self-government or being currently engaged in negotiations to establish such arrangements that include land claim and resource settlements. One example of this arrangement is the establishment of the Territory of Nunavut in 1999 [Indian and Northern Affiars Canada (INAC) 1993].

Just over half of the approximately 100,000 northern residents are Aboriginal and belong to distinct cultural groups including the Yukon First Nations (Yukon), Dene, Métis and Gwich’in (Northwest Territories), and Inuit (Nunavut, Nunavik, the new Inuit land claim area of Nunatsiavut within the region of Labrador and the Inuvialuit Settlement Region of the Northwest Territories). Many of the communities are characterized by an increasingly young and rapidly growing population: 54% of the population of Nunavut is under 15 years of age compared with the national average of 25% (Statistics Canada 2001). Many still experience lower health status than their southern counterparts. For example, life expectancy among Aboriginal people in some regions, such as Nunavik, is as much as 12 years lower than the national average for both sexes (Statistics Canada 2001). In addition, many remote communities are challenged by limited access to health services, lower average socioeconomic status, crowding and poor-quality housing, and concerns regarding basic services such as drinking water quality (Statistics Canada 2001). Despite these challenges, all northern cultures retain a close relationship with the environment and a strong knowledge base of their regional surroundings. Even today, the environment and the country foods that come from the land, lakes, rivers and sea remain central to the way of life, cultural identity, and health of northern Aboriginal people (Van Oostdam et al. 2005). More than 70% of northern Aboriginal adults harvest natural resources through hunting and fishing and of those, > 96% do so for subsistence purposes (Statistics Canada 2001). This strong relationship with their environment plays a critical role in the ability of northern Aboriginal peoples to observe, detect, and anticipate changes in their natural environment.

## Climate Change in Canada’s North

The breadth of scientific research on the Canadian northern environment has grown significantly in recent decades. Scientific research, monitoring, and observations and the knowledge we have acquired from Aboriginal people have resulted in an awareness that changes are taking place. Observed trends vary depending on the region and period analyzed. For example, the western and central Arctic have experienced a general warming over the past 30–50 years of approximately 2–3°C (Weller et al. 2005). This warming is more pronounced in winter months. It is not until the last 15 or so years that this same warming trend, although not to the same extent, has been observed in eastern regions of the Canadian Arctic. Observed impacts associated with these changes include a significant thinning of sea- and freshwater ice, a shortening of the winter ice season, reduction in snow cover, changes in wildlife and plant species’ distribution, melting permafrost, and increased coastal erosion of some shorelines (Cohen 1997; Huntington and Fox 2005; Ouranos 2004; Weller et al. 2005). According to the Arctic Climate Impact Assessment (ACIA 2005) designated climate models, the predictions are for increased warming and precipitation throughout the Canadian Arctic. Annual mean warming in the west is projected to range between 3 and 4°C and upwards of 7°C in winter months. Winter warming is expected to be greatest in the more centrally located areas of southern Baffin Island and Hudson Bay (3–9°C). A 30% increase in precipitation is predicted by the end of the 21st century, with the greatest increases occurring in areas of greatest warming (Weller et al. 2005). The predicted impacts on the environment, regional economies, and people are far reaching. Recent research projects have begun to identify specific local vulnerabilities and risk management measures/adaptation strategies that are already in place or that can be planned (e.g., Berkes and Jolly 2002; Ford et al. 2006; Nickels et al. 2002); however, very little attention has been given to health impacts and adaptations in this region to date.

## Assessing Health Impacts and Vulnerability

Health data series and regional scale assessments in the Canadian North are limited. However, recent qualitative studies examining the potential health impacts of environmental change provide new insights with which to focus research and proactively develop response strategies. They show the need for community participation in filling information gaps and increasing our understanding of factors that enhance or inhibit adaptive capabilities (Furgal et al. 2002; Nickels et al. 2002). The cases we review below present some of these experiences.

### Climate change and health in Nunavik and Labrador

The project Climate Change and Health in Nunavik and Labrador: What We Know from Science and Inuit Knowledge (Furgal et al. 2001) was conducted in the communities of Kuujjuaq, Nunavik (Québec), and Nain, Nunatsiavut (Labrador), in 2000–2001. The project was initiated by members of regional Aboriginal (Inuit) agencies in charge of local environmental health issues in cooperation with a university researcher (C. Furgal, Laval University, Québec City, Québec, Canada). The project was conducted to establish a baseline understanding of the relationship between environmental changes observed in the communities and the potential impacts of these changes on health, as perceived by participants and reported in the health sciences literature.

Nunavik is home to approximately 9,000 Inuit residents living in 14 villages distributed along the coasts of Ungava Bay, Hudson Straight, and the eastern shore of Hudson Bay (Figure 1). In 2005, the autonomous Inuit region of Nunatisavut was established via a tripartite agreement between the federal and provincial governments and the Inuit of Labrador. This region is situated within the mainland boundary of the province of Newfoundland and Labrador. The region is home to approximately 4,800 Inuit living in five coastal communities (Figure 1). Despite recent economic, political, and social changes in the regions of Nunavik and Nunatsiavut over the past decades, residents remain close to their traditions, and many aspects of a land-based traditional lifestyle are still commonly practiced.

To identify potential impacts of observed climate-related changes on health, the project gathered information from various sources. Investigators reviewed the available scientific literature, conducted expert consultations with northern health and environment professionals and researchers, and documentation of Inuit knowledge and perspectives via focus groups with 16 Inuit hunters, elders, and women in the two communities. A process of thematic content analysis was then performed on the qualitative data, and common groups or categories of environmental changes and human impacts were developed (Tesch 1990). This analysis of the collective base of information identified a series of potential direct and indirect health impacts associated with climatic changes observed in Nunavik and Nunatsiavut (Table 1; Furgal et al. 2002).

Most observations and impacts were common between the two regions. For the purposes of the our discussion here to present the scope of changes and impacts observed to date, the results of these two regions are combined. Participants in the two regions identified changes in climatic conditions over the past 10 years not previously experienced or reported in the region. Some changes were identified as having a direct impact on the health of individuals. Respiratory stress was reported among elderly participants and those with decreased respiratory health in association with an increase in summer temperature extremes that now exceed 30°C in both regions. The reported increase in uncharacteristic weather patterns and storm events had significant impacts on travel and hunting/fishing safety. As one focus group participant reported:

it changes so quick now you find. Much faster than it used to. . . . last winter when the teacher was caught out it was perfect in the morning. . . then it went down flat and they couldn’t see anything. . . . Eighteen people were caught out then, and they almost froze, it was bitterly cold. (Nunatsiavut focus group participant, unpublished data, 2001)

Significantly more indirect associations between climate-related changes and health were reported by local residents, northern environment and health professionals, or were found in the pertinent scientific literature (Table 2). For example, warming winter temperatures in the areas around both communities were reported to have changed the timing of ice freeze-up and decreased its thickness and stability. For Inuit communities, sea ice travel is critical for accessing wildlife resources and traveling between communities during winter months. There are anecdotal reports of an increase in the number of accidents and drownings associated with poor or uncharacteristic ice conditions during times of the year that are predictable and typically very safe. More events are reported each year, such as that occurring in 2003 when two young Inuit men went through the ice on their skidoos and drowned near their community as a result of a strange thinning ice phenomenon that was reported to have been “becoming more common in recent years” (Nelson 2003). With a young and increasingly sedentary population spending more time in communities engaged in wage employment and less time on the land, a combination of factors appears to make this group more vulnerable to the climate-related changes being reported in many northern regions today. Moreover, changes in the timing of the ice season are reported to impact the frequency and timing of hunting activities in communities, as indicated by the following comment:

This year and last year, we have been stopped when we were going to go fishing. The ice broke up quickly. We would have gone fishing more in the past. (Nunavik focus group participant, unpublished data, 2001)

The implications of these changes on food security and potential implications on nutritional health among these populations which receive significant energy and nutrient contributions to their total diet from these country foods is only now being investigated. In fact, a number of focused research projects have been initiated with the communities involved in this present study and others in these regions. For example, work on climate and water quality, hunting behavior, women’s health, and emerging and chronic diseases in the North are currently under way.

In general, the impacts identified by local residents in this project were supported primarily by scientific evidence and the published literature, although, in some cases, the effects represented new findings. Many impacts were based on individuals’ experiences in relation to observed climate-related changes in the local area. Other impacts were identified as “potential,” as they were logical extrapolations for residents considering the observed patterns of change in regional climate variables and the perceived relationship between Inuit health and the environment (Tables 1 and 2).

### Inuit community workshops on climate change

In response to growing concern among Inuit communities about environmental changes being observed, the national Inuit organization in Canada, Inuit Tapiriit Kantami, initiated a project in cooperation with regional Inuit organizations and Canadian research institutions to document changes and impacts experienced in communities and to discuss how communities currently are adapting or may adapt in the future. In the first series of workshops in January and February 2002, a research team involving regionally based Inuit representatives visited three of the six communities in the Inuvialuit Settlement Region of the Northwest Territories (Tuktoyaktuk, Aklavik, and Inuvik, Northwest Territories; Figure 1). Community workshops occurred over 2 days in each community, and research team members documented Inuit residents’ observations of environmental changes and the reported effects they were experiencing in association with these changes. At the same time, communities began to identify existing strategies or develop potential adaptation strategies for local-level response (Table 3; Nickels et al. 2002). The processes used for the workshop drew on participatory analysis and planning techniques including Participatory Rural Appraisal (PRA) and Objectives Oriented Project Planning (ZOPP) [Chambers 1997; Deutsche Gesellschaft für Technische Zusammenarbeit (German Agency for Technical Cooperation) 1988].

The communities of the Inuvialuit Settlement Region (ISR) have been observing changes associated with warming in their region for a longer period than those living in the eastern Arctic communities. Changes in the ISR appear more pronounced. For example, increased mean summer and winter temperatures, temperature extremes, an increase in uncharacteristic weather patterns and storm events, a decrease in precipitation, and changes in the characteristics of the ice season similar to those reported in the eastern communities (Furgal et al. 2002) were discussed in ISR community workshops (Nickels et al. 2002). These changes affect the health of individuals and communities, and in some cases communities are already beginning to respond (Table 3).

For example, in association with summer warming, residents are reporting an increase in the number and species of biting flies and insects, including bees. Many residents are concerned because of the potential for spread of disease or potential allergic reactions to stings, as many of these insects have never been seen before in this region. Consequently, a public education process was recommended by workshop participants to inform people about what action could be taken to minimize the risk of being bitten and to alleviate public fear. Currently, little information on these topics exists or is available in the communities (Table 3).

Locally appropriate strategies were suggested to address climate-related impacts on animal distribution and decreased human access to important country food sources (e.g., caribou and geese). Furgal et al. (2002) reported that some people (e.g., Elders and those with limited equipment and financial resources) were challenged in their access to country food species, particularly during fall and spring because of changes in ice conditions, water levels, or shifts in animal migrations. These changes were resulting in increased costs and time associated with traveling longer distances to procure these foods and a decrease in consumption of these items for some members of the community. Because of these problems, it was recommended that a community hunting and sharing program be formalized to ensure access to these food stuffs for all (Table 3).

Currently, more reactive than proactive strategies are in place to adapt to climate-related health impacts in these communities. Changes in hunting behavior, increased investments in equipment or infrastructure (e.g., smoke houses, freezers), and the importance of increased education and information exchange were identified. As in the eastern Arctic communities, these initial workshops have led to the establishment of a variety of projects that address specific issues. Some of these projects will potentially lead to proactive primary adaptations to reduce exposure (Casimiro et al. 2001).

## Understanding the Capacity of Canada’s North for Health Adaptation

A summary of examples of adaptive strategies from the work presented in Table 3 is indicative of the inherently adaptive nature of Inuit society and northern Aboriginal cultures in general (Adger et al. 2003; Nickels et al. 2002; Reidlinger and Berkes 2001). However, the ability to respond varies among communities and regions and is influenced by some common critical factors. The World Health Organization framework for health adaptation (Grambsch and Menne 2003) identifies seven elements that influence vulnerability and adaptation to climate-related health impacts, many of which are applicable to the northern communities discussed here.

The ability to overcome changes in access to or availability of country food resources, which are important for nutritional and sociocultural well-being, is significantly influenced by an individual’s access to economic resources and technology. The ability to invest more in the required tools and equipment for hunting and traveling, or the access to other forms of transportation (e.g., snow machine, four-wheel all terrain vehicle, flat bottom or larger boat) allows individuals to adapt more easily to changing environmental conditions (Duhaime et al. 2002; Ford et al. 2006).

Similarly, the generation and sharing of local or traditional knowledge of regional environments and the relationship between the environment and humans further support this ability to adapt while on the land and safely navigate increasingly dangerous and uncharacteristic conditions. The ability to shift species, alter hunting behaviors, and read environmental cues (e.g., weather prediction, ice safety) all increase hunting and travel safety and success. The importance of this knowledge is gaining recognition among scientific and policy communities (e.g., Huntington and Fox 2005); however, its generation is being challenged locally with shifts toward a more “western lifestyle” involving more time spent in communities engaged in indoor wage-based economic activities and less time on the land (Chapin et al. 2005).

The support provided through institutional or formal arrangements for aspects of traditional lifestyles and health may become increasingly important with climate change in Arctic regions. As many communities begin to represent more pluralistic societies in terms of livelihoods and lifestyles, establishing country food collection, storage and distribution programs, and economic support for the pursuit of traditional activities become important in reducing the vulnerabilities to and enhancing adaptive capabilities for climate-related changes. Also important is the formalization of traditional knowledge documentation and sharing mechanisms through the establishment of such things as community-based ice monitoring programs (Lafortune et al. 2004).

With warming temperatures and the potential for the introduction of new water and foodborne agents and permafrost melting, which threatens built structures in coastal communities, some basic public health infrastructures (e.g., water treatment and distribution, emergency transportation) are increasingly vulnerable. The security of basic public health infrastructure in small remote communities that are already challenged regarding provision of some basic services is a significant determinant of adaptive ability in these locations.

Finally, existing health status issues in Inuit populations (e.g., nutritional deficiencies, increasing rates of diabetes and some cancers associated with shifts toward a more “western diet” and sedentary lifestyle, and rates of respiratory illness) appear to be further exacerbated by changes in local climate. The combination of environmental change, basic health needs, limited economic choices, and shifts in northern society and lifestyle appears to increase vulnerability and limit the ability of some Arctic communities to respond. When many of these factors overlap and the population is already facing some critical health issues, the impact of climate change is greater because of the population’s vulnerability (e.g., small remote communities, with a limited natural and economic resource base).

## Discussion

Indigenous populations are often more vulnerable to climatic changes because of their close relationship with the environment, their reliance on the land and sea for subsistence purposes, the fact that they are more likely to inhabit areas of more severe impact such as coastal regions, often have lower socioeconomic status, are more socially marginalized, and have less access to quality health care services (Kovats et al. 2003). In the public health sector, this combination of the current exposure–response relationship, the extent of exposure, and the possible preventative measures in place creates a vulnerability baseline against which the effectiveness of future policies can be measured via changes in the burden of disease (Ebi et al. 2003). The dialogue approach we present here shows the value of establishing this baseline and engaging Arctic Aboriginal communities on these issues by a process very similar to that outlined by Ebi et al. (2006).

The findings presented in these two small studies are supported by others (e.g., Ford et al. 2006; Krupnik and Jolly 2002). A workshop with Northern health professionals, community leaders, and Aboriginal representatives from across the North reported similar results (Health Canada 2002). Critical issues identified included challenges related to northern home design and a lack of ventilation causing heat stress among elderly on increasingly warm days; impacts to food security because of changes in sea-ice access routes to hunting areas or ice-road stability and effects on reliable transport of market food stuffs; combined impacts on mental health due to reduced ability of individuals to practice aspects of traditional lifestyles; and impacts to infrastructure and threats of community disruption or relocation (Health Canada 2002).

Although a regionally based analysis was not possible with the data available, variations in vulnerabilities and adaptive abilities appear to exist between and within regions on the basis of a number of common factors (see “Understanding the Capacity of Canada’s North for Health Adaptation”; Grambsch and Menne 2003). Similarly, both projects were conducted with Inuit communities, and hence, differences between Arctic cultural groups were not identified. However, as each Aboriginal group is uniquely adapted to its geography and local ecology, it is reasonable to speculate that each group’s socioecologic resilience and adaptive capacity for health issues is similarly unique. Observed climate changes, impacts, and response abilities of Yukon First Nations living in the interior of the western Arctic likely are very different from those of the Inuit communities presented here. It is therefore critical to conduct such assessments locally.

As in other regions of the world, enhancing adaptive capacity can be regarded as a “no regrets” option in the North, as it not only reduces vulnerability but also improves immediate resilience to current day stresses (Yohe and Tol 2002). Strengthening access and availability to country foods throughout the year for communities or increasing public health education associated with environmental causes of disease are such examples. Establishing community freezer and distribution plans will help in addressing current nutritional and other food issues as well as increase the capability of an individual to access safe and healthy foods in the face of environmental changes. Increased knowledge and awareness of environmental causes of disease will address perceived risks and provide valuable information to empower individuals to continue to make healthy decisions.

Both the Nunavik–Nunatsiavut (Labrador) project and the workshops in the ISR are starting points in the collection of information to support community, regional, national, and international processes on climate change. Many new projects have since begun on components of the climate–health relationship in northern communities, and many of these are taking a similarly participatory approach (e.g., ArcticNet 2004). Arctic indigenous peoples have also participated in the international assessment of climate change impacts through their involvement in the ACIA with academic and government researchers (ACIA 2004). This level of engagement and contribution is a significant advance in environmental health impact and vulnerability research. Despite these advances, research on climate and health in northern Aboriginal populations is sparse (Berner and Furgal 2005), and the identification of the impacts on local populations and community adaptations is still in its infancy and requires continued effort with attention to thresholds and limits to adaptation (Berkes and Jolly 2002).

The studies presented here on populations in Canada’s North and a review of other recent research in this region (e.g., ACIA 2005; Ford et al. 2006; Health Canada 2003) identify data gaps that we need to fill and methods that we need to use to increase our understanding of climate and health assessment, vulnerability, and the capacity to adapt in northern Aboriginal communities. They include the following:

### Multiple-scale research and data

Community-based assessments and systematic research must be conducted on the issues of climate change impacts in the North and elsewhere in Canada. Local, regional, and national levels are interconnected in supporting and facilitating action on climate change; thus data at multiple levels and research that link scales to understand these relationships are needed. Fine-scale meteorologic data is required in many northern regions and must be collected in a way that allows the data to be linked to existing and future health data sets. Models of change and impact must be linked with currently used global change scenarios.

### Quality, comparable, standardized data

Innovative approaches to health and climate assessment are needed and should consider the role of sociocultural diversity present among Arctic communities. This requires both qualitative and quantitative data and the collection of long-term data sets on standard health outcomes at comparable temporal and spatial levels. These data must include local observations and knowledge collected using reliable and standardized methods.

### Integrated, interdisciplinary approaches to assessment

Assessments that take a multi-disciplinary approach bringing together health scientists, climatologists, biologists, ecologists, social and behavioral scientists, and policy researchers and include demographic, socioeconomic, and health and environmental data are required to develop an adequate understanding of impacts, vulnerabilities, and capabilities in Arctic communities.

### Increased analysis of historical data

Historical data (climate, health, social, economic) from appropriate locations with climate systems similar to those projected for Canadian northern regions must be used for integrated and geographic analyses of the spread of disease relative to climate variables. These analyses would make efficient use of existing information and increase our understanding of these issues and their interconnected nature.

### Improvement of scenarios and models for health assessment

Developing and improving regional scenarios is needed for areas projected to experience significant impacts, such as the western Arctic. Socioeconomic scenarios to model and project impacts and changes within northern indigenous populations are needed. Such scenarios are currently sparse, poorly developed, and inadequate.

### Conceptual and analytical understanding of vulnerability and capacity

Work is needed at both the conceptual and analytical levels to define and increase our understanding of vulnerability and community health, how best to measure these concepts, and the use of these concepts in making decisions about the health of the community and in risk management. This work should include local knowledge and informal institutions (e.g., cultural sharing networks) to best understand these concepts in Aboriginal communities.

### Enhancement of local capacities to identify, conduct, and analyze data related to climate change and the impacts on health

To ensure success and sustainability of adaptation strategies, development of local and regional monitoring, analytical and decision making capabilities are needed to support cooperative and empowering approaches to research and action.

## Conclusions

In the Canadian North the debate is no longer solely about identifying and predicting effects of climatic change but rather about what can and should be done to adapt, as some communities are already reporting impacts. This research focuses on improving the understanding of the magnitude and timing of the impacts of climate change, how individuals and communities cope with current and predicted changes, and what public institutions should do to actively support adaptation.

There is currently sparse information on the effectiveness of any current strategies for dealing with climate-related or environmental risks to health in the locations described here and in other areas of the country. This lack of information is an important gap in our understanding and ability to assess who, where, and when Canadians may be vulnerable to the effects of climate change. A significant component is the lack of an assessment of the Canadian health sector’s ability at various levels and in various locations to cope with and plan for the impacts of climate change. The cooperative planning, development, and conduct of projects in Inuit communities bringing together scientists, northern environment and health professionals, and community residents and experts, as presented here, has been essential to the success of the projects described in this article. The community-based, dialogue-focused approach has proven valuable in engaging communities and establishing a local baseline for understanding the changes, impacts, vulnerabilities, and the ability to respond at the local scale. Such an approach may very well prove useful in establishing this baseline in other regions.

## Figures and Tables

**Table 1:** Summary of potential direct climate-related health impacts in Nunavik and Labrador.

Identified climate-related change	Potential direct health impacts
Increased (magnitude and frequency) temperature extremes	Increased heat- and cold-related morbidity and mortality
Increase in frequency and intensity of extreme weather events (e.g., storms)	Increased frequency and severity of accidents while hunting and traveling, resulting in injuries, death, psychosocial stress
Increase in uncharacteristic weather patterns	
Increased UV-B exposure	Increased risks of skin cancers, burns, infectious diseases, eye damage (cataracts), immunosuppression

UV-B, ultraviolet B. Adapted from Furgal et al. (2002).

**Table 2:** Summary of potential indirect climate-related health impacts in Nunavik and Labrador.

Identified climate-related change	Potential indirect health impacts
Increased (magnitude and frequency) temperature extremes	Increase in infectious disease incidence and transmission, psychosocial disruption
Decrease in ice distribution, stability, and duration of coverage	Increased frequency and severity of accidents while hunting and traveling, resulting in injuries, death, psychosocial stress Decreased access to country food items; decreased food security, erosion of social and cultural values associated with country foods preparation, sharing, and consumption
Change in snow composition (decrease in quality of snow for igloo construction with increased humidity)	Challenges to building shelters (igloo) for safety while on the land
Increase in range and activity of existing and new infective agents (e.g., biting flies)	Increased exposure to existing and new vectorborne diseases
Change in local ecology of waterborne and foodborne infective agents (introduction of new parasites and perceived decrease in quality of natural drinking water sources)	Increase in incidence of diarrheal and other infectious diseases Emergence of new diseases
Increased permafrost melting, decreased structural stability	Decreased stability of public health, housing, and transportation infrastructure Psychosocial disruption associated with community relocation (partial or complete)
Sea-level rise	Psychosocial disruption associated with infrastructure damage and community relocation (partial or complete)
Changes in air pollution (contaminants, pollens, spores)	Increased incidence of respiratory and cardiovascular diseases; increased exposure to environmental contaminants and subsequent impacts on health development

Adapted from Furgal et al. (2002).

**Table 3:** Examples of environmental changes, effects, and coping strategies/adaptations reported by community residents in the Inuvialuit Settlement Region to minimize negative health impacts of climate change.

Observation	Effect	Coping strategy/adaptation
Warmer temperatures	Not able to store country food properly while hunting; food spoils quicker; less country foods are consumed	Return to community more often in summer while hunting to store food safely (in cool temperatures) Needed: investment of more funds for hunting activities Decrease amount of future hunting and storage with fewer places to store extra meat Needed: re-investment in government-supported community freezer program
Warmer temperatures in summer	Can no longer prepare dried/smoked fish in the same way: “It gets cooked in the heat” Less dried/smoked fish eaten	Alter construction of smoke houses: build thicker roofs to regulate temperature Adapt drying and smoking techniques
Lower water levels in some areas and some brooks/creeks drying up	Decrease in sources of good natural (raw) drinking water available while on the land Increased risk of waterborne illnesses	Bottled water now purchased and taken on trips
More mosquitoes and other (new) biting insects	Increased insect bites Increasing concern about health effects of new biting insects not seen before	Use insect repellent, lotion, or sprays Use netting and screens on windows and entrances to houses Needed: information and education on insects and biting flies to address current perception/fear
Changing animal travel/migration routes	Makes hunting more difficult (requires more fuel, gear, and time) Some residents (e.g., Elders) cannot afford to hunt, thus consuming less country foods	Initiation of a community program for active hunters to provide meat to others (e.g., Elders) who are unable to travel/hunt under changing conditions Needed: financial and institutional support to establish program

Adapted from Nickels et al. (2002).
